# MAGT-toll: A multi-agent reinforcement learning approach to dynamic traffic congestion pricing

**DOI:** 10.1371/journal.pone.0313828

**Published:** 2024-11-18

**Authors:** Jiaming Lu, Chuanyang Hong, Rui Wang

**Affiliations:** 1 School of Business Administration, Southwestern University of Finance and Economics, Chengdu, China; 2 School of Computing and Artificial Intelligence, Southwestern University of Finance and Economics, Chengdu, China; 3 School of Management, Chongqing University of Technology, Chongqing, China; University of Jeddah, SAUDI ARABIA

## Abstract

Modern urban centers have one of the most critical challenges of congestion. Traditional electronic toll collection systems attempt to mitigate this issue through pre-defined static congestion pricing methods; however, they are inadequate in addressing the dynamic fluctuations in traffic demand. Dynamic congestion pricing has been identified as a promising approach, yet its implementation is hindered by the computational complexity involved in optimizing long-term objectives and the necessity for coordination across the traffic network. To address these challenges, we propose a novel dynamic traffic congestion pricing model utilizing multi-agent reinforcement learning with a transformer architecture. This architecture capitalizes on its encoder-decoder structure to transform the multi-agent reinforcement learning problem into a sequence modeling task. Drawing on insights from research on graph transformers, our model incorporates agent structures and positional encoding to enhance adaptability to traffic flow dynamics and network coordination. We have developed a microsimulation-based environment to implement a discrete toll-rate congestion pricing scheme on actual urban roads. Our extensive experimental results across diverse traffic demand scenarios demonstrate substantial improvements in congestion metrics and reductions in travel time, thereby effectively alleviating traffic congestion.

## Introduction

Urban traffic congestion presents a formidable challenge for many cities today, leading to prolonged travel times, exacerbated air pollution, and increasing commuter frustration. In response to this problem, municipalities take corrective actions like congestion pricing, traffic signal control, ramp metering [1]. They are introduced to secure the regularity, the safety of roads, and limit their influence on the environment. In the last several years, reinforcement learning has significantly advanced traffic signal control [2, 3]. By dynamically adjusting signal timings, reinforcement learning can effectively manage traffic flow at intersections, reduce wait times, and alleviate congestion. However, during high-traffic peak hours, signal control alone may be insufficient, making congestion pricing a complementary measure.

Congestion pricing fundamentally diverges from traffic signal control in approach and application. Signal control represents a supply-side management strategy, optimizing vehicular movement at intersections by refining signal timing and phasing. Conversely, congestion pricing exemplifies demand-side management, influencing driver behavior through dynamic toll adjustments. By imposing variable fees on highly congested routes or zones, this strategy aims to internalize the externalities of peak-hour travel, thereby incentivizing travelers to select alternative travel times, routes, or modes. As an economic mechanism, congestion pricing has proven effective in mitigating congestion, reducing traffic volume, and improving traffic flow [4].

Congestion pricing schemes are meticulously designed using various strategic approaches. Dynamic peak-hour pricing models, for example, dynamically calibrate rates in response to fluctuating traffic patterns [5], while fixed-fee structures levy a predetermined charge on vehicles traversing specified congested routes [6]. Additionally, some cities employ cordon-based pricing [7], mandating tolls for vehicles entering designated zones, a strategy that has demonstrated considerable success in urban centers like London [8]. Advanced electronic toll systems, leveraging onboard units and automatic license plate recognition technologies, streamline toll collection processes by eliminating delays from traditional physical infrastructure such as toll booths, thus significantly improving traffic flow [9]. Given the diverse structures of congestion pricing, the topic often invites controversy and is typically restricted to specifically designated regions. To ensure equitable outcomes, transparency in fee structures and an emphasis on societal welfare remain paramount considerations in the implementation of this pricing mechanism [10].

Nevertheless, research on road tolls reveals several significant limitations. Firstly, traditional toll systems typically employ static pricing, meaning toll rates are fixed and cannot adapt to changes in traffic conditions. This approach has limited effectiveness in alleviating congestion as it cannot adequately respond to the dynamic fluctuations in traffic flow. Secondly, model-based congestion pricing schemes require creating models for travelers’ route choices, which often involve complex calculations. For instance, day-to-day models attempt to predict route selection, but their accuracy is often constrained by assumptions and modeling errors [11]. Currently, the most advanced research direction is road tolling methods based on reinforcement learning, which dynamically adjust toll rates to accommodate real-time traffic conditions [12, 13]. However, they face challenges in coordinating control across complex road networks. Additionally, some studies implement route-based tolling [14], but this approach has inherent drawbacks: as the network expands, path enumeration rapidly increases, making precise modeling challenging [15]. Another limitation is that, in the majority of studies, toll rates are typically modeled as continuous variables to facilitate the application of differential calculus. However, discrete toll rates are more comprehensible and distinguishable to travelers in practice. Nevertheless, discrete tolling presents significant challenges to congestion pricing systems, as its lack of differentiability makes optimization using conventional models difficult, resulting in a high-dimensional combinatorial optimization problem.

To overcome these limitations, we propose a multi-agent reinforcement learning solution that treats each toll road as an individual agent. By leveraging reinforcement learning algorithms, it effectively coordinates and optimizes tolling strategies across the entire network. This approach offers enhanced flexibility and adaptability, allowing efficient operation in more complex traffic networks while addressing many of the shortcomings inherent in traditional methods. Our primary contributions are as follows: (1) We investigated a congestion pricing scheme for the road network, focusing on a discrete toll system during peak hours. Due to the exponential expansion of the agents’ action space, we introduced multi-agent reinforcement learning techniques to address this challenge. (2) We developed a multi-agent congestion pricing algorithm named MAGT-toll, which uses transformer modules to construct an agent-by-agent sequential decision-making framework. By incorporating agent position and structural encoding, our algorithm effectively manages complex road networks, enhancing adaptability across various traffic scenarios. (3) Compared to benchmark methods, our approach excels in several key metrics. MAGT-toll efficiently handles road tolls in dynamic traffic demand scenarios at a network scale, demonstrating significant potential in reducing congestion.

The remaining content arrangement: The **Related works** section further supplements the literature on road tolls and introduces the work of multi-agent reinforcement learning and graph transformers. The **Problem definition** section outlines the congestion pricing simulation environment presented in this paper and details how it is defined as an MDP problem. The **Methodology** section provides a detailed description of the model used in this paper. The **Experimental results and analysis** section delves into the experimental details and analyzes the results, while The **Discussion** section concludes the paper.

## Related work

The literature review for this study was conducted through a systematic search approach. We initially performed extensive searches across multiple databases, including IEEE Xplore, ACM Digital Library, SpringerLink, and Google Scholar, using search terms such as “dynamic congestion pricing,” “multi-agent reinforcement learning,” and “graph transformer.” The selection focused primarily on top-tier journals and conference papers published within the last decade, ensuring that the cited works are both highly representative and at the forefront of the field. Additionally, we employed citation tracking, further exploring the references cited in the core papers to ensure a comprehensive and well-rounded literature base.

### Dynamic congestion pricing

Traditional methods of congestion pricing, such as fixed and area-based pricing, primarily rely on model-driven approaches. The Dynamic Congestion Pricing (DCP) model, specifically the Single Bottleneck Model introduced by [4], innovated dynamic pricing systems by incorporating the concept that charges for a given time period should reflect the equilibrium cost of waiting time in an unpriced network. [16] further advanced this concept by providing an approximate analytical formula for congestion pricing in general networks, based on Kuhn-Tucker optimality conditions. [17] analyzed vehicle volumes entering and exiting networks to propose an optimal, model-based pricing strategy. [18] tackled urban traffic network pricing using Simulation-Based Optimization (SBO) with Macroscopic or Network Fundamental Diagrams (MFD/NFD). They found that Regression Kriging (RK) is particularly effective in managing complex scenarios due to its noise-filtering capability and rapid convergence. The model-based congestion pricing scheme necessitates accurate modeling, which makes it challenging to apply in traffic environments characterized by complexity and change. Therefore, [19] proposed Δ-tolling, a simple adaptive pricing scheme that relies only on travel time observations and two regulatory parameters. This approach is suitable for the entire road network, allowing frequent updates without the need for complex traffic or demand models. While feedback control methods are easy to implement, they may require multiple adjustments under complex traffic conditions. Simultaneously, [20] employed a dynamic congestion pricing method based on reinforcement learning to mitigate traffic congestion during morning rush hours. This approach dynamically adjusts pricing using real-time traffic data to alleviate congestion. For instance, the Deep-RL framework developed by [21] utilized a partially observable Markov decision process (POMDP) to balance revenue maximization with minimizing total travel time. The regional road pricing model employing an edge-based Graph Convolutional Network, as proposed by [22], demonstrated superior performance over conventional methods during real-world testing on Singapore’s road network. Research indicates that dynamic pricing strategies effectively reduce congestion and optimize road usage, but opportunities for enhancement remain. For example, while most existing research scenarios employ continuous pricing rates, discrete rates align more closely with practical applications, as does the coordinated control of multi-agent networks for congestion pricing at the network level.

**Multi-agent reinforcement learning** (MARL) is used to address scenarios where multiple agents learn and interact with each other within an environment. Two common architectures exist: Centralized Training, Decentralized Execution (CTDE): In CTDE, agents share information during training, usually guided by a central role providing feedback. This approach can significantly accelerate the learning process and is particularly effective for tasks requiring collaboration [23]. During execution, agents operate independently, relying only on their individual data. This architecture is frequently used in team-based scenarios like sports team simulations or multi-robot coordination [24]. Fully Decentralized Architecture: Here, agents train and execute entirely independently without central control or shared information. Each agent learns through interactions with the environment and other agents [25, 26]. This architecture is suitable for competitive or non-cooperative settings [27]. Recent studies have introduced a new multi-agent architecture based on the advantage decomposition theorem [28, 29]. In this agent-by-agent sequential decision-making framework, agents act according to a specific order, and the sum of their local advantages aligns with the global advantage. This allows agents to update their policies independently without disrupting the overall advantage. [30] combined the encoder-decoder structure from the transformer model with the advantage decomposition theorem to propose the multi-agent transformer (MAT), currently the most advanced MARL model. While multi-agent reinforcement learning has been extensively proven in traffic signal control tasks, there is still limited work concerning congestion pricing.

### Graph transformer

The graph transformer model is designed for handling graph-structured data. The original Transformer architecture was created for sequential data, like natural language. In the classical Transformer, the self-attention mechanism calculates relationships between nodes globally [31]. However, in graph-structured data, node and edge relationships are more complex, requiring the Graph Transformer’s attention mechanism to incorporate structural information, such as node distances and edge features [32, 33] introduced centrality and spatial encoding to capture node importance and spatial relationships. Centrality encoding assigns importance based on node degrees, while spatial encoding uses the shortest path distance to convey structural information. Edge information is crucial when processing graph data. [34] incorporated an edge channel into the traditional Transformer, handling edge features and dynamically updating this information across layers. [35] categorized positional and structural encoding methods as global, local, and relative. [36] provided a comprehensive review of graph transformer models from an architectural design perspective, systematically evaluating their effectiveness across graph tasks and exploring future research directions. The Graph Transformer is well-suited for modeling traffic networks due to its ability to manage complex graph structures and identify relationships between nodes. This is essential for traffic networks as they are intricate systems of roads, intersections, and vehicle flows. This paper draws from recent Graph Transformer research and integrates it with multi-agent reinforcement learning to tackle congestion pricing under network coordination.

## Problem definition

The key mathematical symbols in this paper and their descriptions are listed in Table 1.

**Table 1:** Summary of mathematical symbols.

Symbol	Description
*r*	A route in the traffic network
*l*	A road in the route *r*
*L*_*l*_	Length of the road *l*
*v*_*l*_	Average speed on the road *l* during time interval Δ*t*
*M*_*l*_	Toll rate for the road *l*
*ϵ*	Price coefficient representing the trade-off between time and monetary cost
Δt	Time interval for toll rate updates
O	*Joint state set*: The set of all joint states at time *t*, $\{o_{i}^{t}\}\in O$, where $\left\{o_{i}^{t}\right\}$ is the observation of agent *i* at time *t*
A	*Joint action set*: The set of all joint actions at time *t*, $\{a_{i}^{t}\}\in A$, where $\left\{a_{i}^{t}\right\}$ is the action chosen by agent *i* at time *t*
R	*Joint reward set*: The set of all joint rewards within decision interval Δt, where each reward corresponds to the outcome of joint actions taken by the agents
$A_{i_{j}}^{\pi }\left(O,a_{i_{1}:j-1},a_{i_{j}}\right)$	*Advantage function*: Describes the relative advantage of agent *i*_*j*_ taking action $a_{i_{j}}$ given the actions $a_{i_{1}:j-1}$ of other agents
**RW**	*Random Walk Matrix*
**p**_RWPE,*i*_	*Positional Encoding of node i*: Calculated using Random Walk Positional Encoding (RWPE) to capture the global position of node *i* in the graph
$h_{i}^{\left(l+1\right)}$	*Feature vector of node i at layer l* + 1

### Description of the congestion pricing environment

We have established a congestion pricing simulation environment using the CityFlow microscopic traffic simulation platform [37] to emulate vehicle movement within a traffic network. The platform takes traffic networks and routes as input: traffic networks define the interconnection of intersections and roads, while routes detail each vehicle’s departure time, starting point, and destination. In this study, we expanded the platform to incorporate a congestion pricing mechanism that imposes fees on designated roads to influence drivers’ route choices, encouraging them to plan routes based on travel costs.

In this simulated environment, we focus on three core elements:

**Traffic signals**: To ensure that the traffic signal control strategy does not interfere with the assessment of congestion pricing effects, this study employs a fixed-time signal control. The signals automatically switch phases every 20 seconds in a predetermined sequence.

**Toll roads**: Specific roads in the traffic network have been designated for toll implementation in this study. The toll-rate dynamically adjusts according to traffic conditions, updating at regular intervals Δt. The rates are set as discrete values, incrementally increasing in the sequence [2, 4, 6, 8, 10], expressed in standard currency units (e.g., U.S. dollars).

**Drivers**: Drivers choose their route based on the principle of minimizing the total travel cost at the time of departure. Before entering the traffic network, each driver identifies what they perceive to be the optimal route, taking into account current toll rates and traffic conditions. Once a route is selected, drivers maintain their chosen route without making subsequent adjustments during the journey. This assumption reflects the behavior of many real-world drivers, who typically make route decisions based on known information at the start of their journey rather than frequently adjusting their route while driving. The adoption of this simplified assumption is motivated by the inherent uncertainty in driver preferences and the focus of our study on analyzing the impact of tolling mechanisms on traffic flow in response to demand changes. By streamlining the driver decision-making process, we mitigate the complexity introduced by behavioral variability, thereby enabling a more focused evaluation of tolling mechanisms. The travel cost for a specific route *r* is defined by the following equation:

$$\begin{array}{c} \text{Travel}\;\text{cost}\;\text{for}\;\text{route}\;r=\sum_{l\in r}(\frac{L_{l}}{v_{l}})+\epsilon M_{l} \end{array}$$ (1)

**where**:

- *l* represents the individual roads traversed by route *r*,
- *L*_*l*_ is the length of road *l*,
- *v*_*l*_ is the average speed on road *l* during the time interval Δ*t*,
- *M*_*l*_ is the toll rate for road *l*,
- *ϵ* is the price coefficient, representing the interchangeability of money and time costs. In this paper, we set *ϵ* to 15, with detailed discussions on parameter settings found in the **Sensitivity analysis of the price coefficient** section.

**Fig 1:**
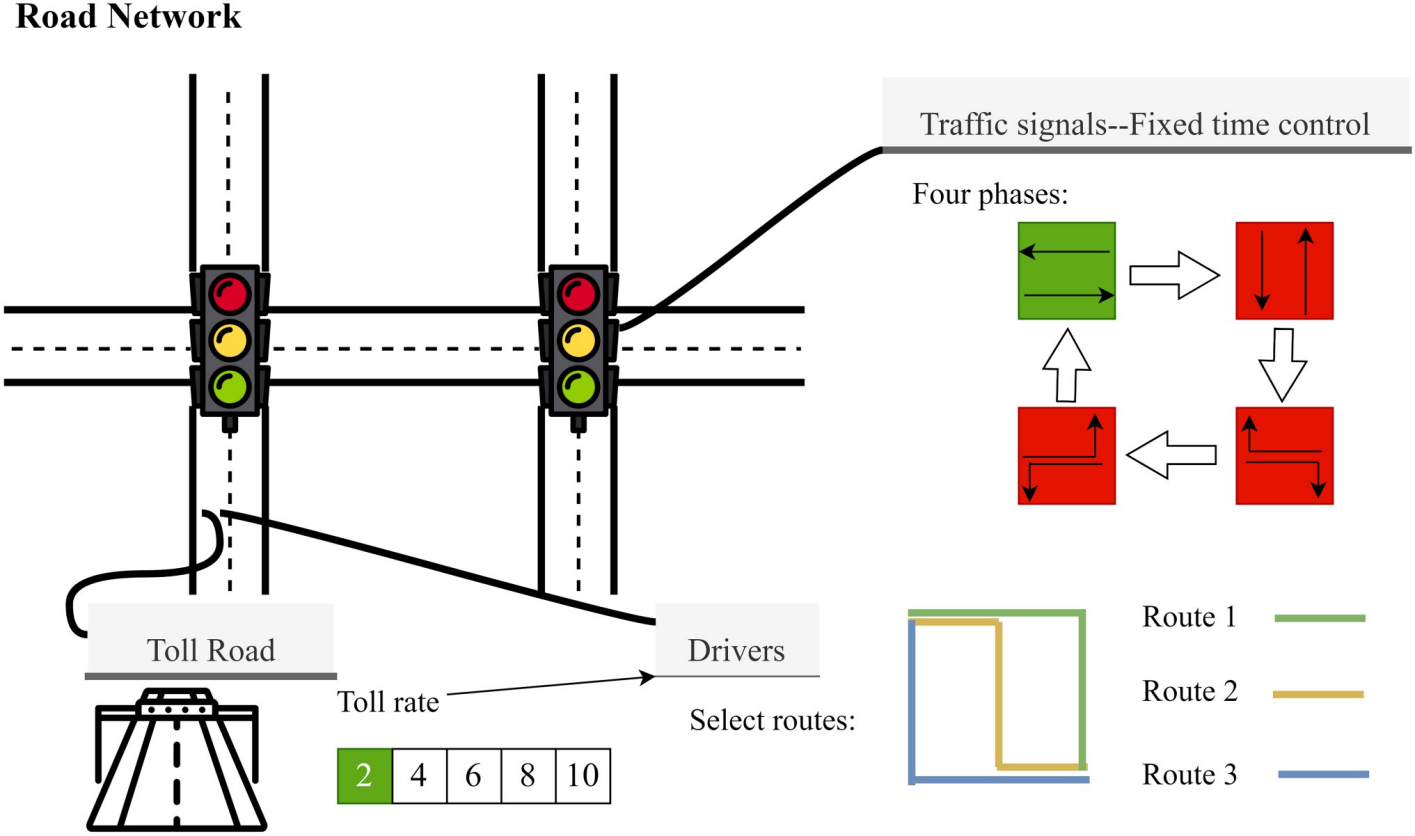
The congestion pricing simulation environment.

The congestion pricing simulation environment is illustrated in Fig 1.

### The markov decision process of congestion pricing

We define congestion pricing as a Markov decision process:

**State**. In the context of congestion pricing, the state is defined as the number of vehicles that arrived at the road during the previous interval, Δt, together with the current vehicle queue. This design allows the agent to understand historical traffic changes and respond to current conditions. In this work, the state has been refined to include the condition of each lane.

**Actions**. In this study, the action space is defined as discrete, comprising five distinct adjustment options, each representing a specific modification to the toll rates. Such adjustments could influence drivers’ route choices and road utilization rates. While most research models toll rates as continuous values to facilitate derivation and ensure theoretical perfection, in practical application, continuous rates might affect commuters’ judgment of route selection and lack interpretability. Traditionally, congestion pricing under continuous rates could be addressed with single-agent reinforcement learning. However, in settings with discrete rates, the problem of exponential growth in the dimensionality of the action space arises, where the size of the action space expands to 5^n^ as the number of tolled roads becomes *n*. Consequently, to tackle this challenge, this study introduces the use of a multi-agent system, establishing independent decision-making agents for each tolled road.

**Rewards**. The ultimate goal of implementing congestion charging is to reduce the overall travel time across the transportation network. However, as travel time encompasses the entire process from a vehicle’s entry to its exit, it is challenging to observe accurately at a specific point in time and thus cannot be used as real-time feedback in the model-environment interaction. In this paper, the reward for a given road *l* is defined as the negative average queue length of vehicles on that road during the decision interval Δt. The reward *R*_*l*_ is given by:

$$\begin{array}{c} R_{l}=-\frac{1}{N}\sum_{i=1}^{N}q_{l}\left(t_{i}\right) \end{array}$$ (2)

where *q*_*l*_(*t*_*i*_) represents the queue length on road *l* at time *t*_*i*_, and *N* is the total number of observations within the interval Δt. During this interval, the queue length *q*_*l*_(*t*_*i*_) is measured every 20 seconds. This reward function incentivizes the agent to minimize queue lengths, thereby improving traffic flow and reducing congestion.

Existing edge computing technology is capable of supporting real-time traffic monitoring and management. Recent studies indicate that a multi-layer sensing system based on the Internet of Things (IoT) can transmit and process vast amounts of traffic data in real time, thereby supporting complex dynamic pricing systems [38]. Although traditional traffic monitoring sensors, such as loop detectors, radar, and photoelectric sensors, are effective in monitoring traffic flow and estimating vehicle queue lengths, they often involve high installation and maintenance costs and lack scalability. To address these limitations, we propose the adoption of computer vision-based technologies for capturing lane-level traffic data. By integrating these technologies with deep learning algorithms such as Faster R-CNN and YOLO, accurate vehicle detection and tracking can be achieved under various environmental conditions [39]. A lane-level traffic estimation method based on deep learning has been introduced [40], utilizing high-resolution video data from cameras to estimate the number of vehicles in each lane. To optimize performance, cameras should be placed above high-traffic areas, such as overpasses or tall lampposts, ensuring a wide view of multiple lanes and minimizing the risk of occlusion by other vehicles or obstacles. However, vision-based traffic monitoring technology still faces some common challenges in terms of accuracy. Key factors affecting the precision of visual monitoring include camera angles, weather, and lighting conditions. Improper camera positioning may lead to data occlusion, and lighting conditions during nighttime or in strong light environments can also impact detection accuracy. To overcome these limitations, traditional sensors can be integrated to correct data, and this hybrid sensing strategy can enhance data reliability and accuracy while maintaining cost-effectiveness. Overall, integrating IoT architecture with deep learning technology ensures that the current sensing techniques meet the demands of dynamic pricing systems in complex traffic environments.

## Methodology

**Fig 2:**
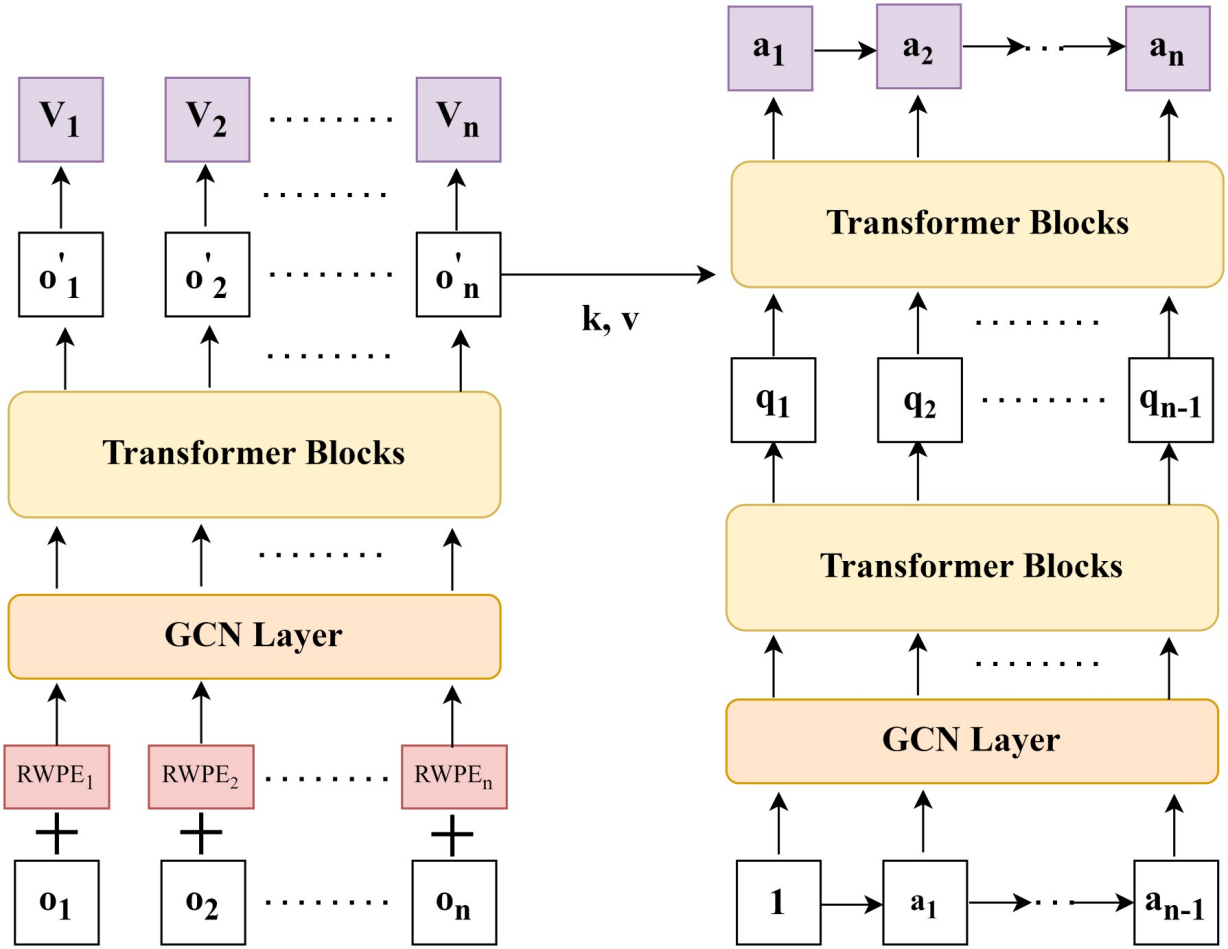
Schematic of the model architecture.

We propose MAGT-toll—a model for multi-agent congestion pricing. The system’s architecture is presented in Fig 2, and it consists of an encoder and a decoder that work in a similar way to an Actor-Critic framework. The encoder assesses the agents’ observations, adding random walk encoding features to each agent’s state, and uses a GCN (Graph Convolutional Network) module to learn the input for the transformer block. The decoder acts as the actor during decision-making. We assign a fixed value, such as 1, as the initial action input, treating it as an isolated node when encoding actions via the GCN. To determine each agent’s next action, the decoder takes the preceding agent’s action sequence and the abstract observation representation provided by the encoder, then outputs the desired action.

### Preliminaries: Multi-Agent Transformer

The MAGT model is based on the MAT (Multi-Agent Transformer) architecture and introduces a novel approach [30]. Unlike traditional methods where all agents make decisions simultaneously, MAT uses a sequential decision-making strategy, handling decisions agent by agent. The core concept of MAT leverages the modeling capabilities of large sequence models to transform multi-agent decision-making into a sequential modeling problem. This allows mapping agent observation sequences to action sequences. As a result, there’s no longer a need to consider all potential joint action combinations at once, as actions can be selected for each agent gradually and sequentially.

In MAT, the state observed by agent *i* at time *t* is denoted as $o_{i}^{t}$, and the agent selects action $a_{i}^{t}$ based on the current policy *π*. The agent’s action selection can be expressed as the following sequential decision process:

$$\begin{array}{c} a_{i}^{t}=\text{Decoder}\left(\text{Encoder}\left(o_{1}^{t},\ldots ,o_{n}^{t}\right),a_{1}^{t-1},\ldots ,a_{i-1}^{t-1}\right)) \end{array}$$ (3)

The Multi-Agent Advantage Decomposition Theorem, utilized by MAT, outlines the advantages conferred by employing multiple agents. This process can be represented by the following formula. In a system consisting of *n* agents, each agent *i* observes the state *o*_*i*,*t*_ and takes action *a*_*i*,*t*_ at time step *t*. The theorem states that for any sequence of agents *i*_1:*n*_, the collective advantage $A_{i_{1}:n}^{\pi }\left(O,a_{i_{1}:n}\right)$ can be expressed as:

$$\begin{array}{c} A_{i_{1}:n}^{\pi }\left(O,a_{i_{1}:n}\right)=\sum_{j=1}^{n}A_{i_{j}}^{\pi }\left(O,a_{i_{1}:j-1},a_{i_{j}}\right) \end{array}$$ (4)

$A_{i_{j}}^{\pi }\left(O,a_{i_{1}:j-1},a_{i_{j}}\right)$ denotes the advantage of agent *i*_*j*_ in taking action $a_{i_{j}}$ given the actions $a_{i_{1}:j-1}$ of other agents. MAT adopts an encoder-decoder architecture where the encoder learns the representation of joint observations, and the decoder autonomously generates the actions of each agent in a self-regressive manner. The encoder aims to minimize the experiential Bellman error, with its objective function denoted as *L*_Encoder_:

$$\begin{array}{c} L_{\text{Encoder}\;}\left(\phi \right)=\sum_{j=1}^{n}(R(O_{t},A_{t})+\gamma {\bar{V}}^{\phi }({\hat{o}}_{i_{j}}^{t+1})-V^{\phi }({\hat{o}}_{i_{j}}^{t}){)}^{2} \end{array}$$ (5)

The function ${\bar{V}}^{\phi }$ pertains to the target network, serving to stabilize the training process, while *V*^*ϕ*^ represents the value function outputted by the encoder. The objective of the decoder is to minimize the PPO objective function *L*_*Decoder*_(*θ*), which is defined as follows:

$$\begin{array}{c} L_{Decoder}\left(\theta \right)=-\sum_{j=1}^{n}\sum_{t=0}^{T-1}\text{min}(\frac{\pi_{i_{j}}^{\theta }\left(a_{i_{j}}^{t},|{\hat{o}}_{1:j}^{t},a_{1:j-1}^{t}\right)}{\pi_{i_{j}}^{\theta_{\text{old}}}\left(a_{i_{j}}^{t},|{\hat{o}}_{1:n}^{t},a_{1:j-1}^{t}\right)},1\pm \epsilon ){\hat{A}}_{t}^{j} \end{array}$$ (6)

$\pi_{i_{j}}^{\theta }$ represents the policy output by the decoder, while ${\hat{A}}_{t}^{j}$ refers to the estimate of the joint advantage function.

### Multi-Agent position and structure encoding

Before addressing position and structure encoding, a graph network is constructed where roads serve as nodes. Specifically, if the tail node of one road is connected to the head node of another road, a relationship is established. The connectivity between two roads is determined by the lanes at the intersection. For reverse lanes of a road, a connection exists only if there is a “U-turn” type lane; otherwise, no connection is present.

**Using Random Walk Positional Encoding (RWPE)** [41]. In graph neural networks, positional encoding aims to assist the model in comprehending the global position of each node within the overall graph structure. This understanding becomes particularly crucial when nodes have similar local neighborhoods but play different roles globally. To address this issue, a random walk-based positional encoding approach has been proposed. In this approach, a random walk on the graph is defined as a sequence of vertices that are randomly selected from the neighbors of the current vertex. This method not only captures the concept of movement across the graph but also provides the node’s neighborhood with diverse and random sampling over varying distances.

A random walk on a graph is characterized by a procedure in which an adjacent node is randomly selected from a specified starting node, with subsequent selections proceeding iteratively from each new node. The process is encapsulated in the Random Walk Matrix, denoted as **RW**, where **RW** = **AD**^−1^. In this formulation, **A** represents the adjacency matrix of the graph, and **D** denotes the degree matrix.

The RWPE can be calculated as follows:

$$\begin{array}{c} {\text{p}}_{\text{RWPE},i}=[{\text{RW}}_{ii},{\text{RW}}_{ii}^{2},\ldots ,{\text{RW}}_{ii}^{k}] \end{array}$$ (7)

where **p**_RWPE,*i*_ denotes the positional encoding of node *i*. Here, **RW**_*ii*_ represents the probability that node *i* remains at its initial position after a single-step random walk, and ${\text{RW}}_{ii}^{k}$ is the probability of node *i* remaining in place after *k* steps. The adjacency matrix **A** defines the graph’s structure, where **A**_*ij*_ = 1 indicates the presence of an edge between nodes *i* and *j*, and 0 otherwise. The degree matrix **D**, a diagonal matrix, has each diagonal element **D**_*ii*_ signifying the degree of node *i*-the number of edges connected to node *i*. The random walk matrix **RW** specifies the transition probabilities, with **RW**_*ij*_ indicating the likelihood of moving from node *i* to node *j* in one step. The parameter *k* signifies the number of steps considered in the random walk, employed in calculating the autoregressive probability of node stability over *k* steps.

Through this method, unique positional encodings can be generated for each node, capturing their global positions within the graph. This assists graph neural networks in better understanding and utilizing the structural information of the graph.

To integrate the agents’ observations and positional encodings, we concatenate the feature vectors of both encodings, thereby crafting a comprehensive representation of the agents’ features:

$$\begin{array}{c} H_{i}^{0}=\left[o_{i}⊕{\text{p}}_{\text{RWPE},i}\right] \end{array}$$ (8)

This embedding can be employed within the GCN framework by initializing node representations with positional embeddings, followed by proceeding with standard graph convolution operations.

**Structural Encoding Using GCNs** [42]. The concatenated feature vector, *x*_*i*_, is fed into the GCN layer for further feature extraction and learning. In the GCN layer, the connected structure among nodes is utilized to update the representations of node features, thereby enhancing the structural information within node features. In GCNs, the feature update of node *i* can be achieved through the following formula:

$$\begin{array}{c} h_{i}^{\left(l+1\right)}=\sigma (\sum_{j\in N(i)\cup \{i\}}\frac{1}{\sqrt{\text{deg}(i)\text{deg}(j)}}W^{\left(l\right)}h_{j}^{\left(l\right)}) \end{array}$$ (9)

In the model, N(i) represents the set of neighboring nodes of node *i*, deg(*i*) denotes the degree of node *i*, *W*^(*l*)^ is the weight matrix for the *l*-th layer, $h_{j}^{\left(l\right)}$ is the feature vector of neighboring node j at the *l*-th layer, and *σ* is the nonlinear activation function RELU. By integrating the positional encodings generated from random walks with observational data, followed by GCN processing, the model effectively combines the positional information of nodes with observational data, enhancing the agent’s perceptual abilities and decision-making quality in the environment.

As detailed in Algorithm 1. This algorithm, termed MAGT-toll, is designed to optimize traffic flow and reduce overall travel time by dynamically adjusting toll rates based on real-time traffic conditions.

**Algorithm 1**: MAGT-toll for multi-agent road congestion pricing

**Input**: Batch size *B*, number of agents *n*, episodes *K*, steps per episode *T*

**Output**: Updated encoder and decoder parameters *ϕ* and *θ*

 1. Initialize: Encoder {*ϕ*_0_}, Decoder {*θ*_0_}, Replay buffer B

 2. **for** *k* = 0, 1, …, *K* − 1 **do**

  **for** *t* = 0, 1, …, *T* − 1 **do**

   Collect the observations of all agents $O_{t}$ from the environment

   Compute positional encodings $p_{t}^{i}$ using (RWPE)

   Concatenate observations and positional encodings: $H_{t}^{i}=\left[o_{t}^{i}⊕p_{t}^{i}\right]$

   Apply GCN layer to concatenate vectors: $H_{t}^{i}\leftarrow \text{GCN-layer}\left(H_{t}^{i}\right)$

   Generate representation sequence ${\hat{o}}_{t}^{i}$ by feeding $H_{t}^{i}$ to the encoder

   Input ${\hat{o}}_{t}^{i}$ to the decoder

   **for** *j* = 0, 1, …, *n* − 1 **do**

    Input $a_{t}^{i_{0}},\ldots ,a_{t}^{i_{j}}$ and infer $a_{t}^{i_{j+1}}$ with the auto-regressive decoder

   **end**

   Execute joint actions $a_{t}^{i}$ in environments and collect the reward $R_{t}$

   Insert $\left(O_{t},A_{t},R_{t}\right)$ into B

  **end**

  Sample a random minibatch of *B* steps from B

  Generate $V_{\phi }\left({\hat{o}}_{t}^{i}\right)$ with the output layer of the encoder

  Calculate *L*_Encoder_(*ϕ*) with Eq (5)

  Compute the joint advantage function $\hat{A}$ based on $V_{\phi }\left({\hat{o}}_{t}^{i}\right)$ with GAE

  Input ${\hat{o}}_{t}^{i},a_{t}^{i}0,\ldots ,a_{t}^{in-1}$, generate $\pi_{\theta }^{i}$ with the decoder

  Apply GCN layer to decoder inputs: $a_{t}^{i}\leftarrow \text{GCN-layer}\left(a_{t}^{i}\right)$

  Calculate *L*_Decoder_(*θ*) with Eq (6)

  Update the encoder and decoder by minimizing *L*_Encoder_(*ϕ*) + *L*_Decoder_(*θ*) with gradient descent

   **end**

## Experimental results and analysis

### Experimental setup

**Fig 3:**
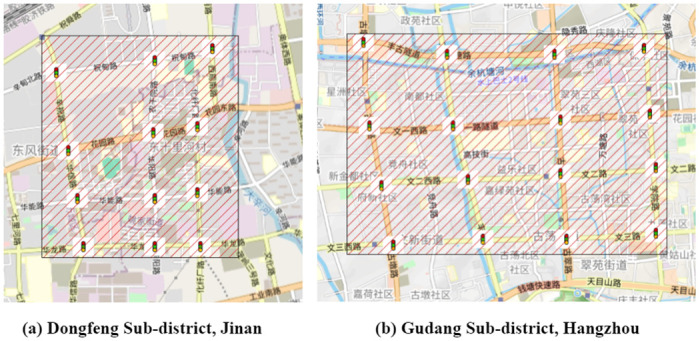
Congestion pricing zones of Jinan and Hangzhou’s traffic networks.

To simulate urban traffic scenarios, this study utilizes the Cityflow platform [37] and focuses on selected road networks from Dongfeng Street in Jinan City and Gudang Street in Hangzhou City. The Jinan network is a 3x4 intersection grid with 62 roads, 34 of which are within zones controlled by 12 traffic signals and designated as congestion-priced roads. The Hangzhou network, a 4x4 intersection grid, comprises 48 roads, 32 of which are within zones regulated by 16 traffic signals and also designated as congestion-priced roads. Fig 3 illustrates the congestion pricing zones for both networks, with specific network parameters detailed in Table 2. These road networks are based on those presented in the study by [43].

**Table 2:** Parameters of road networks in Jinan and Hangzhou.

Parameters	Jinan	Hangzhou
Number of Traffic Lights	12	16
Number of Toll Roads	34	48
Total Number of Roads	62	80
Area (KM^2^)	8	20
Low-Medium-High Vehicle Arrival Rate (veh/s)	[0.5, 0.75, 1.0]	[0.7, 0.9, 1.2]

The duration of the experiment was set to 2 hours (7200 seconds), with a decision time interval of 20 minutes (1200 seconds). The experimental design encompassed three distinct traffic scenarios, simulating ‘low,’ ‘medium,’ and ‘high’ levels of vehicle flow. Since congestion pricing is predominantly implemented during peak periods, we reflected this reality by setting the tolling period to 2 hours. Additionally, the model was trained and evaluated under ‘low’ and ‘medium’ traffic demand scenarios. This approach served two purposes: enhancing the model’s adaptability to varying demand levels, and assessing the necessity of implementing road tolls during non-peak periods. The generation of traffic demand scenarios was based on the assumption that vehicle arrival rates follow a Poisson distribution, with arrival rates for the two networks under the three scenarios specified in Table 2. Vehicle routes were generated according to the original origin-destination (OD) pairs of the randomized network.

### Benchmarks

In this paper, we explore a variety of road tolling algorithms to assess and compare their impacts on traffic flow and revenue generation. In addition to the previously mentioned MAT and MAGT-toll methods, we have also considered the following approaches:

**1. No-toll**: This strategy involves no toll collection whatsoever. Specifically, the toll rates for all roads are set to zero, simulating an unregulated natural state of traffic, thereby serving as a baseline for comparison.
**2. Adaptive-toll**: This method draws inspiration from the delta-toll algorithm proposed by [19]. Originally designed for continuous tolling, our adaptive-toll has been modified for discrete toll-rate scenarios. It adjusts toll rates based on the traffic volume at the end of each time interval. Roads are ranked from highest to lowest traffic volume, and toll rates are assigned according to lane numbers. For the Jinan network, lanes 1 to 7 are charged 10 units, lanes 8 to 14 are charged 8 units, and so forth up to lane 34, thereby facilitating dynamic management and incentivizing congestion reduction. Similarly, for the Hangzhou network, the 48 toll roads are divided into segments of [8, 10, 10, 10, 10] lanes, with charges assigned accordingly.
**3. EGCN-toll**: This approach utilizes an Edge-based Graph Convolutional Network to represent the global state of traffic flow. It is optimized through a framework of CTDE [22].

In the experimental part, these tolling methods will be evaluated and compared based on travel time, which is defined as the average travel time for all vehicles from their entry to the conclusion of the simulation. The hyperparameters for MAGT are detailed in Table 3, and they are adopted from the settings used in the original MAT algorithm paper [30].

**Table 3:** Hyperparameters of MAGT-toll.

Parameters	Values
Learning rate	0.0005
Ppo epochs	5
Number of batches for ppo	1
Ppo clip rate	0.2
Gamma	0.99
Number of transformer blocks	1
Number of attention heads	1
Number of gcn layers	1
Embedded dimensions	64

### Model training and overall performance

**Fig 4:**
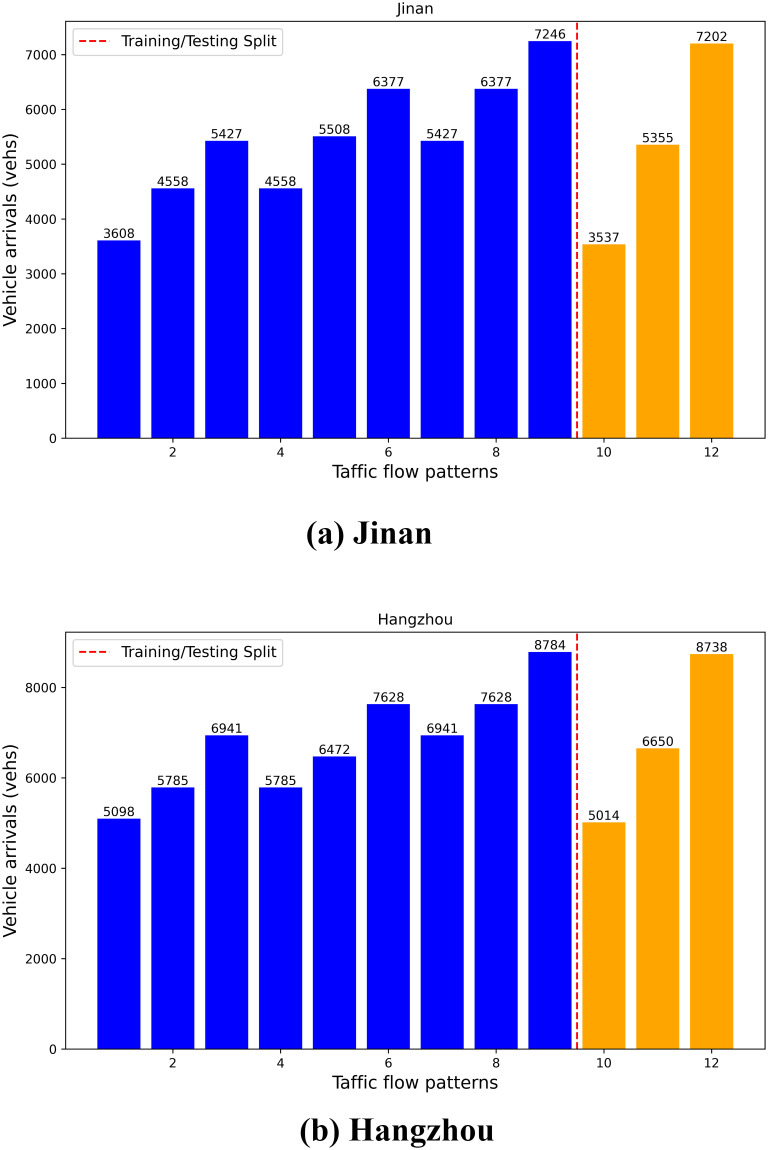
Training and testing scenarios of Jinan and Hangzhou’s traffic networks.

In this paper, we trained three learning-based models (EGCN-toll, MAT, MAGT) using a hybrid traffic demand scenario. By training across various scenarios, we effectively enhanced the models’ generalization ability. For the two-hour decision-making window established in this study, we designed three traffic flow patterns—low, medium, and high—each corresponding to a specific mode per hour. Through permutations and combinations, a total of nine traffic demand scenarios were generated for training. During model evaluation, we regenerated the two-hour continuous ‘low-medium-high’ traffic demand scenarios based on the parameters in Table 2. Fig 4 illustrates the cumulative vehicle arrivals in both the training and testing scenarios.

Each learning-based model underwent 200 episodes of training, with each episode randomly selected from nine different training scenarios. Each model was initialized with five distinct random seeds and trained five times. The models were evaluated based on the results of these repeated experiments, with average rewards and travel time assessed under different random seeds in the test scenarios. Tables 4 and 5 present the comprehensive evaluation results on the road networks of Jinan and Hangzhou.

**Table 4:** Overall performance comparison on the Jinan network.

Scenarios/Methods	no-toll	adaptive-toll	EGCN-toll	MAT	MAGT-toll
**Rewards (vehs)**					
**Low**	\	\	-147.47	-147.73	-147.53
**Medium**	\	\	-939.39	-936.8	-923.66
**High**	\	\	-2143.39	-2202.26	-2116.86
**Average**	\	\	-1076.75	-1095.59	-1061.88
**Average travel time (seconds)**					
**Low**	728.29	560.03	555.51	553.33	554.98
**Medium**	1430.07	1085.37	1076.29	1078.88	1055.66
**High**	2162.44	2027.01	1942.03	1943.63	1915.66
**Average**	1440.26	1224.13	1191.27	1191.94	1175.43

**Table 5:** Overall performance comparison on the Hangzhou network.

Scenarios/Methods	no-toll	adaptive-toll	EGCN-toll	MAT	MAGT-toll
**Rewards (vehs)**					
**Low**	\	\	-167.93	-173.53	-168.22
**Medium**	\	\	-365.06	-359.46	-336.33
**High**	\	\	-670.99	-665.79	-634.07
**Average**	\	\	-401.32	-399.59	-379.54
**Average travel time (seconds)**					
**Low**	991.35	929.45	928.61	929.95	928.32
**Medium**	1273.43	1185.47	1188.11	1186.66	1177.56
**High**	1711.22	1642.75	1641.82	1639.53	1621.54
**Average**	1325.33	1252.55	1252.84	1252.04	1242.47

From Tables 4 and 5, it can be observed that the MAGT-toll consistently outperforms other methods across different traffic flow conditions on both the Jinan and Hangzhou networks. On the Jinan network, compared to the no-toll scenario, MAGT-toll reduces the average travel time by 23.90%, 26.22%, and 11.42% in low, medium, and high demand scenarios, respectively. Similarly, on the Hangzhou network, MAGT-toll reduces travel time by 6.35%, 7.53%, and 5.24% in low, medium, and high demand scenarios, respectively. The performance of MAGT-toll remains superior in the average demand scenarios on both networks, demonstrating its robustness across different environments.

Comparing MAGT-toll with the MAT baseline on the Jinan network, MAGT-toll decreases the average travel time by approximately 16.51 seconds, a reduction of 1.39%. On the Hangzhou network, the reduction is approximately 9.57 seconds, or 0.76%. Although these improvements might seem modest, they become more significant under peak traffic conditions. For instance, in high demand scenarios, MAGT-toll reduces the average travel time from 1943.63 seconds to 1915.66 seconds on the Jinan network, and from 1639.53 seconds to 1621.54 seconds on the Hangzhou network. These reductions are particularly noteworthy in highly congested environments, where even small gains in efficiency can lead to substantial overall improvements in traffic flow.

The enhanced performance of MAGT-toll is largely attributed to its integration of agent position encoding and structural encoding, which improves the algorithm’s ability to identify and respond to dynamic congestion patterns. This capability allows for a more effective distribution of traffic across the network, reducing congestion levels and improving travel times, especially during periods of high traffic demand.

In contrast, the adaptive tolling method (adaptive-toll) shows commendable efficacy on the Hangzhou network but does not outperform MAGT-toll. The results suggest that while real-time toll adjustments can improve traffic conditions, MAGT-toll’s advanced use of graph neural networks and location-aware mechanisms offers a superior approach for handling the complexities of real-world traffic scenarios.

The EGCN-toll, which employs a graph neural network for centralized training and decentralized execution, also performs well, particularly in low-demand scenarios on both networks. However, its decentralized execution approach results in a less coordinated response during peak traffic, leading to performance that lags behind MAGT-toll in medium and high-demand scenarios. This is especially evident on the Jinan network, where the performance gap widens as demand increases, highlighting the advantage of the multi-agent sequential decision-making framework used by MAGT-toll.

Overall, the results across both networks confirm the effectiveness of the MAGT-toll algorithm in managing complex traffic conditions. The algorithm’s enhancements over MAT, particularly in terms of agent collaboration and encoding strategies, allow it to consistently outperform other methods, especially under high traffic demand. These findings suggest that MAGT-toll has significant potential for practical applications in managing complex traffic networks, where optimizing travel times and reducing congestion are critical goals.

### Statistical significance analysis of experimental results

**Fig 5:**
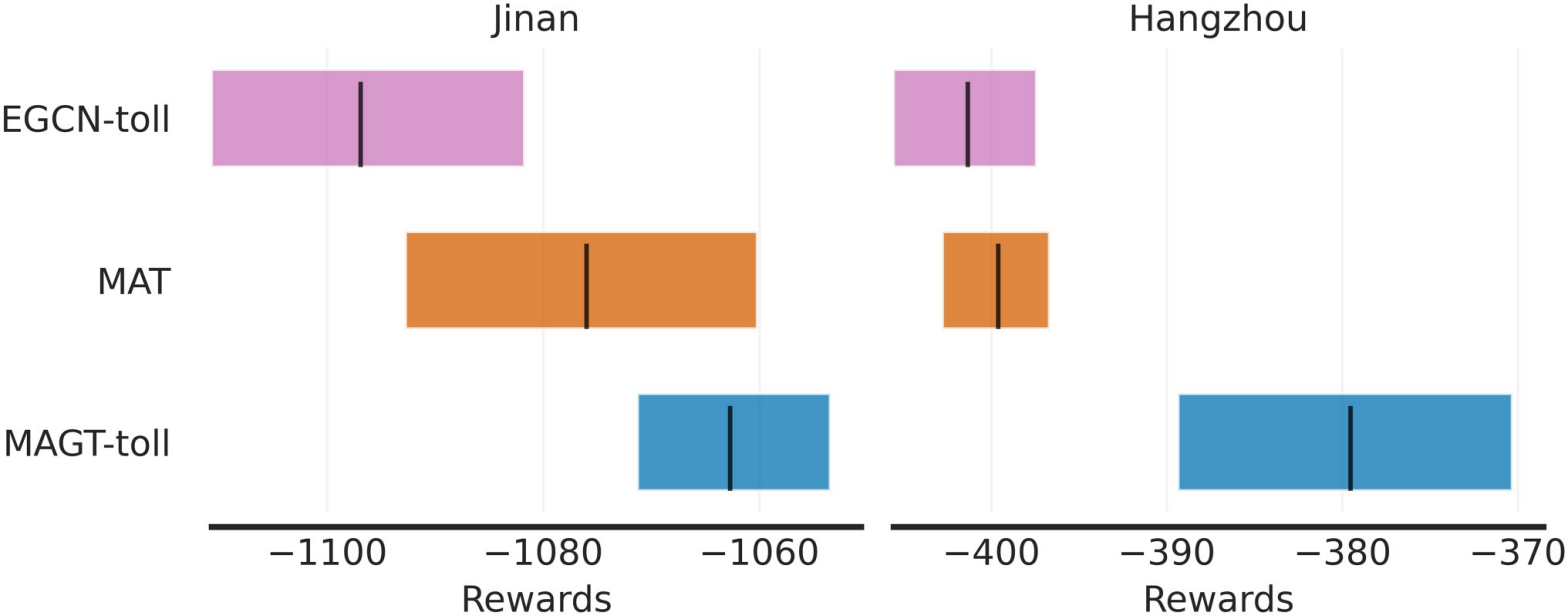
Statistical significance analysis for rewards.

**Fig 6:**
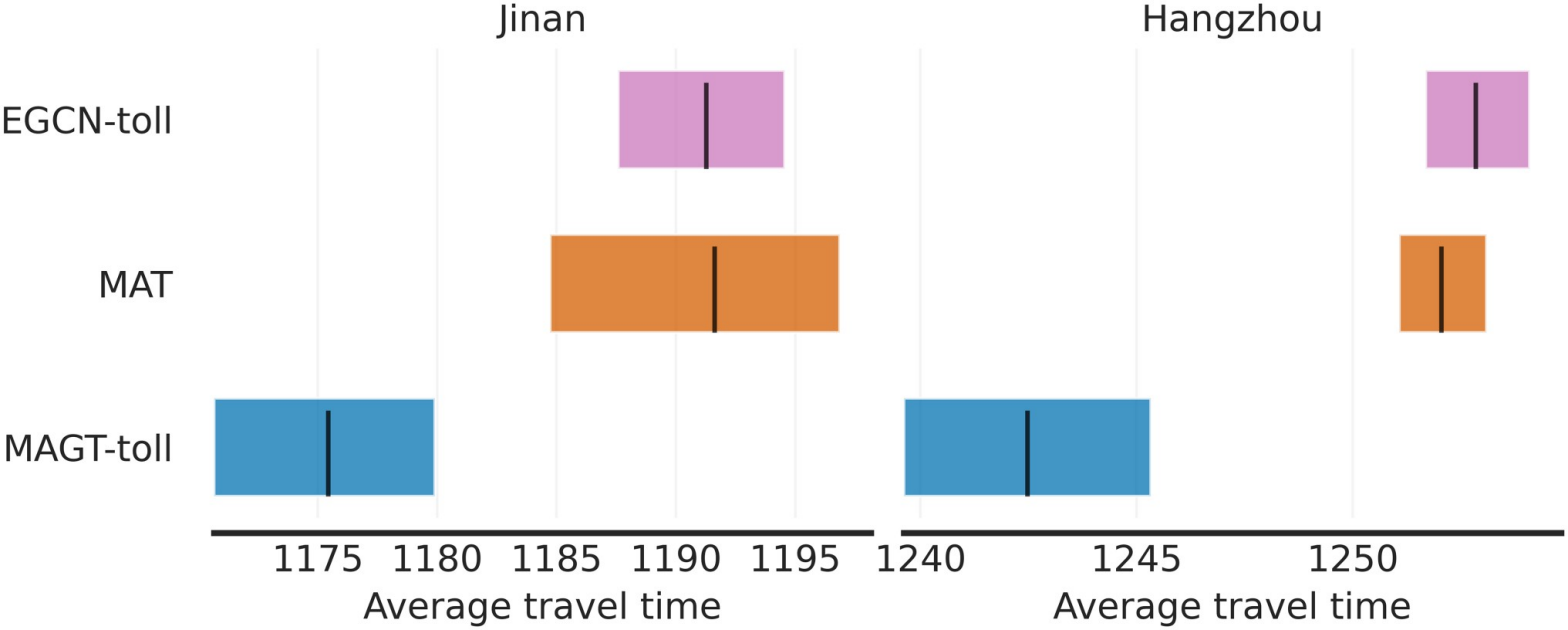
Statistical significance analysis for average travel time.

We conducted a rigorous statistical significance analysis using stratified bootstrap confidence intervals, inspired by the methodology described in [44]. This method provides a robust alternative to traditional p-value-based testing, particularly when the number of experimental runs is limited. We trained the MAGT-toll model and baseline models (EGCN-toll, MAT) using five different random seeds and evaluated their performance across low, medium, and high traffic scenarios on the Jinan and Hangzhou networks. Mean performance metrics and confidence intervals were computed to account for variability across different tasks and random seeds. This approach ensures that the observed improvements are both statistically significant and reliable, even with a limited number of runs. Figs 5 and 6 illustrate the comparison of the rewards and average travel time across the Jinan and Hangzhou networks, respectively, using stratified bootstrap confidence intervals.

On both networks, MAGT-toll consistently outperformed EGCN-toll and MAT across all scenarios. The confidence intervals for MAGT-toll are significantly tighter, indicating less variability and a higher likelihood that the observed differences are genuine. For the Jinan network, the reward intervals show that MAGT-toll’s improvement over MAT is particularly pronounced in the medium- and high-demand scenarios, with minimal overlap in their confidence intervals. Similarly, in the Hangzhou network, the confidence intervals for rewards confirm that MAGT-toll’s performance is consistently better across all demand levels, with the largest margin observed in the high-demand scenario.

The analysis of average travel time reveals that MAGT-toll reduces average travel time more effectively than both EGCN-toll and MAT, particularly under high-demand conditions on both networks. On the Jinan network, the travel time reduction achieved by MAGT-toll is statistically significant, as indicated by the non-overlapping confidence intervals with those of MAT in the high-demand scenario. In the Hangzhou network, while the differences are smaller, the confidence intervals still indicate a consistent improvement, especially in the medium- and high-demand scenarios.

### Sensitivity analysis of the price coefficient

The determination of the price coefficient is a critical aspect of tolling strategies in transportation networks. Ideally, these coefficients should reflect real-world conditions and user behavior, which are often determined through surveys and studies conducted by transportation planning authorities. However, in the context of this study, we have explored a range of price coefficients to observe their effect on the average travel time, ensuring the selected coefficient aligns with the objectives of minimizing travel time while maintaining traffic flow efficiency.

The experiments were conducted using the MAGT-toll algorithm across two distinct road networks—Jinan and Hangzhou. We varied the price coefficient values across a range of 5, 10, 15, 20, and 25, and recorded the corresponding average travel times.

**Fig 7:**
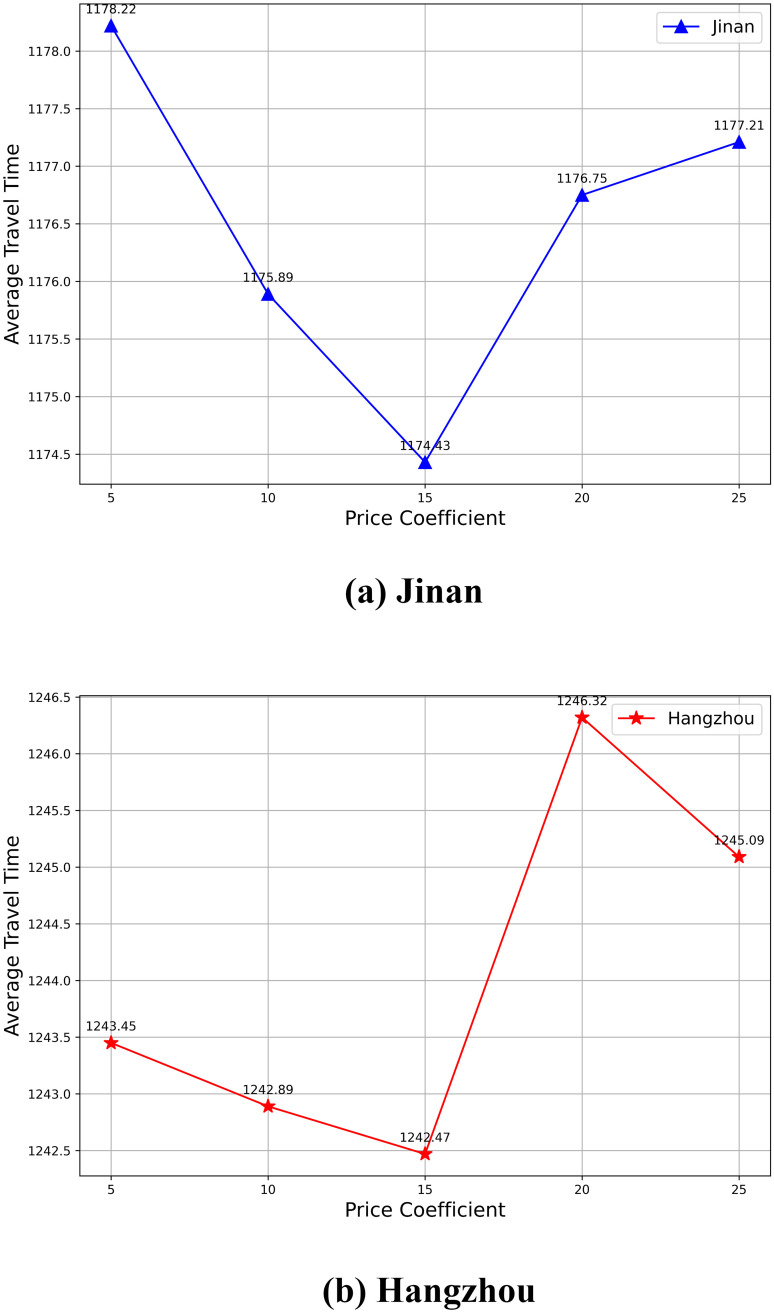
Comparison of average travel time across different price coefficients.

The results in Fig 7 show a consistent pattern across both networks. As the price coefficient increases from 5 to 15, the average travel time decreases. Specifically, in the Jinan network, the travel time decreases from 1178.22 seconds at a coefficient of 5 to a minimum of 1174.43 seconds at a coefficient of 15. Similarly, in the Hangzhou network, the travel time decreases from 1243.45 seconds at a coefficient of 5 to a minimum of 1242.47 seconds at a coefficient of 15.

However, as the coefficient increases beyond 15, the average travel time begins to rise again in both networks. This trend suggests that while moderate toll rates (represented by coefficients between 10 and 15) are effective in optimizing traffic flow and reducing congestion, higher coefficients can lead to inefficiencies, potentially due to over-tolling. Conversely, a coefficient of 5 may be too low to effectively influence traveler behavior, as the perceived cost impact is minimal and insufficient to deter congestion.

### Analysis of specific roads

**Fig 8:**
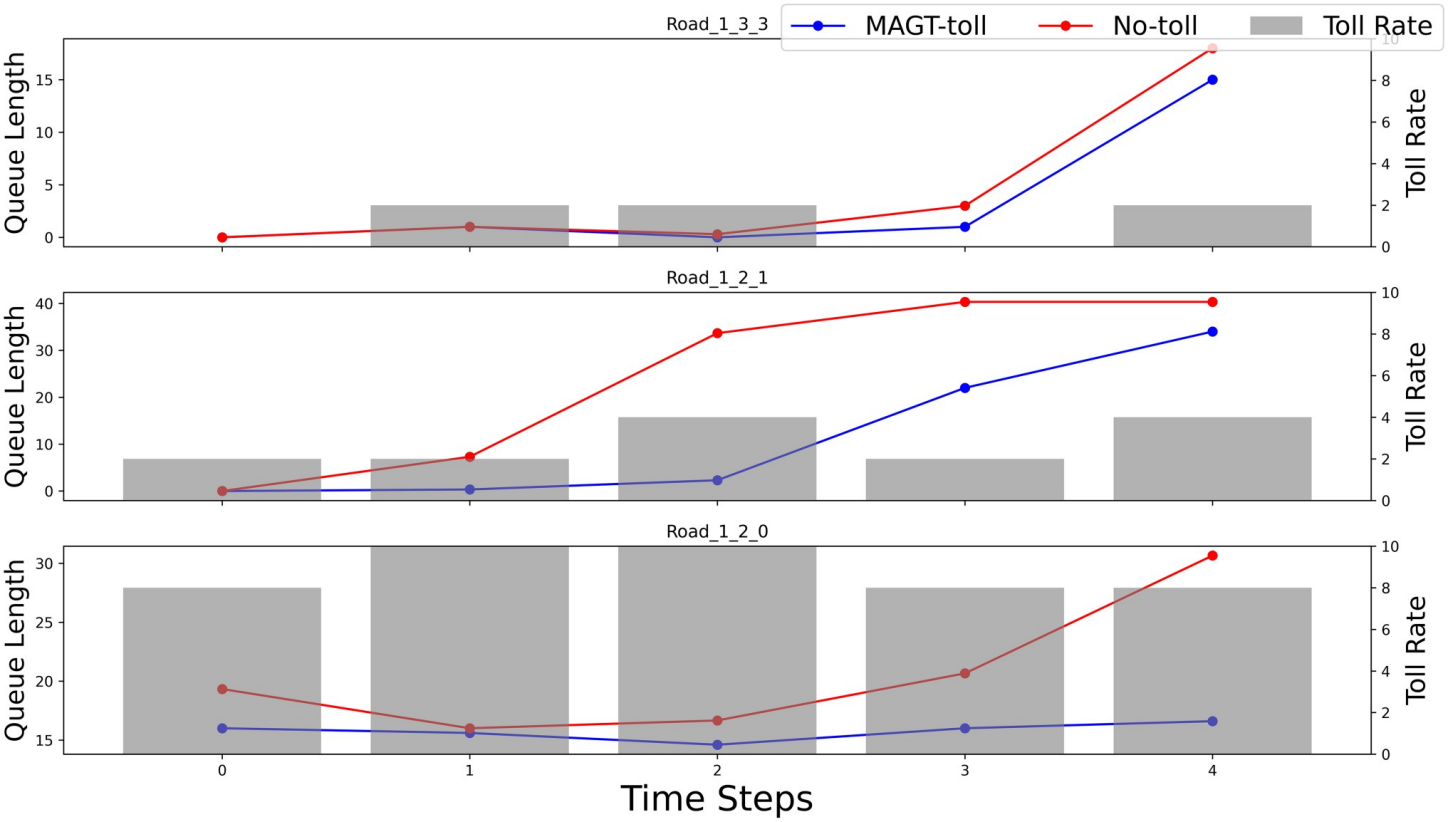
Comparative analysis of the MAGT-toll algorithm and the no-toll situation on selected roads.

To gain a deeper comprehension of how toll mechanisms alleviate traffic congestion, this study selected roads with varying congestion levels under a medium-demand traffic scenario: “road_1_3_3” (relatively unobstructed), “road_1_2_1” (moderately congested), and “road_1_2_0” (congested), to demonstrate the efficacy of the algorithm. As illustrated in Fig 8, we compared the vehicle queue lengths between the MAGT-toll algorithm and the no-toll situation.

Upon implementing the MAGT-toll algorithm on the notably congested roads “road_1_2_1” and “road_1_2_0”, there was a significant reduction in vehicle queue lengths, indicating that the tolling mechanism effectively alleviated traffic congestion on these routes. Specifically, the congestion-pricing system optimized vehicle route selection by adjusting toll rates, thereby dispersing traffic flow and reducing congestion levels. In contrast, for the relatively less busy “road_1_3_3”, there was little change in queue lengths between the tolled and no-toll scenarios during the initial phase of the experiment, with queue lengths under tolling occasionally being slightly shorter. This suggests that in low-congestion areas, the tolling mechanism might temporarily attract more vehicles to choose that route, causing a minor increase in congestion in the short term. However, in the long run, the road “road_1_3_3” did not experience more severe congestion. This reflects the capability of the MAGT-toll algorithm to dynamically adjust toll-rate based on overall traffic flow changes, achieving long-term congestion mitigation through coordinated multi-agent network management.

## Discussion

In this study, we proposed MAGT-toll, a novel dynamic traffic congestion pricing model that integrates multi-agent reinforcement learning with a transformer architecture. This model was designed to tackle the complexities associated with discrete-rate congestion pricing schemes in urban traffic networks. By transforming the decision-making process into a sequential modeling problem, MAGT-toll enhances the adaptability of traffic flow management and network coordination through advanced positional and structural encoding mechanisms. Our comprehensive experiments, conducted across various traffic scenarios, including simulations on selected urban areas in both Jinan City and Hangzhou City, demonstrated the superior performance of MAGT-toll in reducing travel times and alleviating congestion. The model consistently outperformed other benchmark methods, such as no-toll, adaptive-toll, EGCN-toll, and its foundational version MAT, particularly under high-demand scenarios. The enhanced performance of MAGT-toll is further validated by our rigorous statistical significance analysis, which confirms the reliability and robustness of the observed improvements. This success is largely attributed to the model’s advanced sequential decision-making architecture and the collaborative mechanisms between agents, enabling effective management of complex traffic networks under dynamic congestion pricing conditions.

Despite the commendable performance exhibited by MAGT-toll in addressing dynamic congestion pricing in this study, there are several issues that could be further explored and enhanced. 1. Multi-objective optimization: In addition to reducing travel times and congestion, future endeavors could consider environmental impact, economic benefits, and social advantages as part of a multi-objective optimization problem. 2. Policy-making support: exploring how the MAGT-toll algorithm could be applied to assist in policy-making, providing data support and decision-making recommendations to urban planners and traffic managers.
